# Information and Communication Technology Use on New Generation Teachers’ Job Satisfaction and Psychological Emotion

**DOI:** 10.3389/fpsyg.2022.941218

**Published:** 2022-06-21

**Authors:** Pinglei Xu, Ying Jiang

**Affiliations:** ^1^School of Management, Shanghai University, Shanghai, China; ^2^School of Management, Guilin University of Aerospace Technology, Guilin, China

**Keywords:** new generation teachers, job satisfaction and psychological emotion, information and communication technology, efficacy, error aversion culture

## Abstract

Despite the growing trend of Information and communication technology (ICT), little is known about its impact on job satisfaction and the mechanisms through which ICT operates. This study aimed to investigate new generation teachers’ ICT use on efficacy and job satisfaction. We conducted a cross-sectional survey on 234 new generation teachers in China. The Results revealed that ICT use had a positive direct impact on job satisfaction of new generation teachers. Teachers’ efficacy plays a partial mediating role and error aversion culture moderates the relationship between ICT use and teachers’ efficacy. This study complements our attempt to understanding the effect, mechanism and boundary condition of ICT use on new generation teachers’ work outcomes. It also provides a new direction for studying ICT use on employees belonging to other generations.

## Introduction

Job satisfaction is a positive emotional experience toward employee job estimation (Locke, 1976; Spagnoli et al., 2012). Previous studies found that employee characteristics and working conditions influence job satisfaction (Judge et al., 2002; Tsai and Huang, 2008). As a new feature of current jobs, Information and Communication Technology (ICT) has greatly changed the way employees work (Kellogg et al., 2020). ICT refers to a technology which can access information by multiple communication means including the Internet, wireless networks, mobile phones as well as other communication media (Berg-Beckhoff et al., 2017). With the growing use of ICT in the workplace, employees have to struggle with new working conditions. Some scholars argued that ICT use may increase excessive workload pressure and anxiety about technology replacing human labor (Tarafdar et al., 2010, 2014; Srivastava et al., 2015), thus causing negative emotions. From the job characteristics perspective, ICT use reduces job satisfaction and job performance (Venkatesh et al., 2010).

However, these studies ignore employee characteristics. Employees belonging to different age-groups may have different views on job satisfaction and experience different emotions due to the changing work way. For example, some may consider working at home through ICT as it may improve work-family balance (Cousins and Robey, 2015). The Person-job fit theory posits that employee job satisfaction depends on the correspondence of individual and environmental attributes (Yu, 2009; Wang et al., 2020). When work demand matches individual’s abilities or individual needs fit work supply, individuals perceive more meaning in their work which increases work engagement (Tims et al., 2016; Van Vianen, 2018). New generation teachers may experience more job satisfaction while using ICT in work. Because they are born and grown up in the era of internet, they can easily adapt to ICT work demands. ICT use may fulfill their need for exploring new things, breaking rules and other psychological needs (Gursoy et al., 2008). Therefore, the ICT use of new generation teachers will promote their job satisfaction according to the person-job fit theory.

Meanwhile, the mechanism of ICT use on job satisfaction may have an effect through teachers’ efficacy. Efficacy refers to a teacher’s judgment on his or her personal ability to make students engaged in learning and achieve desired results, even for students who lack motivation and have difficulties in learning (Tschannen-Moran and Hoy, 2001). Peng and Mao (2015) found efficacy mediated person-job fit to job satisfaction. ICT use causes less stress and burnout, making new generation teachers more confident to acquire teaching resources through ICT. Teachers’ efficacy may promote teachers’ teaching engagement, goal setting and ambition level (Klassen and Chiu, 2010). ICT use may increase new generation teachers’ efficacy, increasing their sense of achievement, thereby increasing their job satisfaction.

In addition, organization culture may influence ICT use in teaching. Error Aversion culture means organization does not accept mistakes, and members in the organization need to cover up mistakes and bear pressure and negative personal image after making mistakes (van Dyck et al., 2005). As errors are inevitable in the application of ICT, new generation teachers may benefit form error communication and analysis. But an error aversion culture may create tension of ICT use, accordingly decreasing the efficacy of new generation teachers in ICT use. Based on the above considerations, the research explored the effect and mechanism between ICT and job satisfaction among new generation teachers from the perspective of personal-job fit, and further investigated error aversion culture’s influence on the above process.

In comparison with former studies, the current research has made contributions in the following aspects. First, it can enrich ICT use research by introducing personal-job fit theory explain ICT use on job satisfaction. Further, it reveals the mechanism by investigating the mediated role of teachers’ efficacy. Second, it expends the boundary conditions of ICT use by examining school culture and explaining how error aversion culture moderates the relationship between ICT use and teachers’ efficacy.

## Theory and Hypotheses

### Information and Communication Technology Use and Job Satisfaction Literature Review

The use of ICT in the workplace has become a daily routine, especially during the COVID-19 outbreak. ICT significantly affects employees’ working way and outcomes. Most researchers have focused on factors that influence the acceptance of technology, including knowledge, experience and beliefs (Pelgrum, 2001; Paraskeva et al., 2008; So et al., 2012). However, with few exceptions (e.g., Venkatesh et al., 2010; Wang et al., 2020), less attention has been paid to how employee’s ICT use influence their job satisfaction.

There are two perspectives toward the effect of ICT use, technostress and job characteristic model. From the technostress perspective, ICT user causes employees’ negative reactions such as stress, burnout and anxiety (Tarafdar et al., 2010, 2014). Torres (2021) found that use of ICT leads to psychological exhaustion and negative affect which further reduces their job satisfaction. Based on the job characteristics model, the effect of ICT use on employee job satisfaction depends on employees perceiving ICT use as a resource or a demand. If ICT use is perceived as a resource, it represents technological diversity, which may improve employee’s work efficiency or work motivation (Golden, 2006, 2007; Venkatesh et al., 2010). For example, the plagiarism detection system eases teachers’ reading of students’ work (Zaher et al., 2020). ICT use also increases individuals’ flexibility for managing their work and home roles, leading to less burnout and improved job satisfaction (Ninaus et al., 2021). Cousins and Robey (2015) found that mobile workers who use smartphones, laptops and other technologies in their work have taken advantage of technological affordances to manage their work-life boundaries. Otherwise, ICT as a job demand require continuous efforts of employees, resulting in stress and negative mood, which reduces job satisfaction. But these studies only emphasized on technology or work feature, ignoring that job satisfaction may be the result of matching job demand and employee characteristics.

### Application of Person-Job Theory

The Person-job fit theory suggest that good fit between individuals and job leads to positive working outcomes such as job satisfaction, organization commitment (Kim et al., 2009; Wang et al., 2020). Person-job fit includes two aspects, the first is demands and ability fit which means the knowledge, skills and ability of employees adapt to the demands of the job; the second is need and supply fit which means employee needs, desires and preferences can be met by the supply of job (Edwards and Cooper, 1990; Jansen and Kristof-Brown, 2006; Kim et al., 2009). Kristof-Brown et al. (2005) using the meta-analysis method showed that person-job matching has a positive correlation with job satisfaction and organizational commitment.

New generation teachers whose aged between 22 and 43 years (Kehrli and Sopp, 2006; Borges et al., 2010) are the main participants of information technology application projects in primary and secondary schools of China. Unlike the previous generations, new generation teachers who grew up with the Internet, have a high acceptance and adaptability to new technology use in the work field. Instead of accepting a standard answer, they want to explore the underlining reasons by themselves and challenge manager’s authority (Kehrli and Sopp, 2006; Gursoy et al., 2008). They prefer autonomy to accomplish their jobs, and feel more satisfied when they participate in job decision making (García et al., 2019).

Based on the person-job fit theory, this manuscript argues that ICT use of new-generation teachers can positively affect their job satisfaction. The information-based teaching environment requires teachers to have the initiative and adaptability of ICT. Teachers should use general software and network teaching platforms to acquire, process, and make digital education resources. New generation teachers generally have a high level of computer knowledge (Kehrli and Sopp, 2006). They can easily apply new technology to acquire educational resources and feel less stress and burnout toward the ICT requirements of school. In addition, new generation teachers who like to explore new things have a strong creative and innovative spirit (Gursoy et al., 2008). ICT use offers them the opportunity to improve traditional teaching methods and experiment with innovative teaching ideas. Verkijika (2020) emphasized that mobile apps with simplicity characteristics meet user’s expectation and enhance user satisfaction. Their psychological needs are met in the teaching environment of ICT use. Shim and Kim (2020) found that dialog support of App Siri can increase users’ positive effect and satisfaction. Through ICT use, new generation teachers may achieve personal-job fit by matching demand and capabilities as well as need and supply, which may increase job satisfaction. Thus, Hypothesis 1 is proposed.

H1: New-generation teachers’ ICT use positively affects their job satisfaction.

### Teachers’ Efficacy as a Mediator

We propose that ICT use can promote teacher efficacy of new generation teachers. According to the personal-job fit theory, when the characteristics of individuals and jobs match, individuals experience less burnout (Edwards and Billsberry, 2010) more engagement in work. Research shows the negative correlation between burnout and self-efficacy, because burnout caused by ICT use makes employees feel incapable of completing their work (Maslach et al., 2001). For the new generation teachers, the matching of ICT use and personal characteristics will reduce burnout. Correspondingly the match will promote new generation teachers’ ICT use to improve students’ learning interest and increase their sense of efficacy.

Efficacy of new generation teachers has a positive impact on job satisfaction. Studies showed that teachers with a high sense of efficacy are able to better plan and organize their classroom (Tschannen-Moran and Hoy, 2001). At the same time, teachers with a high sense of efficacy and willingness to embrace novel thoughts and methods can better satisfy students’ needs (Guskey, 1988; Tschannen-Moran and Hoy, 2001), because new generation teachers who have a higher efficacy can enhance students’ interest in learning and clear students’ doubts. Thus, their job satisfaction may increase from students’ progress. Alegre et al. (2016) found that efficacy enhances job satisfaction by reducing classroom management stress.

Combined with H1, this manuscript argues that new generation teachers’ ICT use promotes job satisfaction through teacher efficacy. Therefore, we propose our second hypothesis as follows:

H2: Teacher efficacy plays a mediating role in the impact of new generation teachers’ ICT use on job satisfaction.

### Error Aversion Culture as Moderator

Culture is an important factor that influences the relationship between person and job (Chuang et al., 2015). This study focuses on school culture and analysis its influence on new generation teachers’ psychological needs and ICT behavior. Teachers exploring ICT in teaching may make errors. Some scholars argue that organizations have an error aversion culture; members perceived error experience as a negative event to be avoided. This will hinder communication, analysis, and learning from errors (van Dyck et al., 2005; Guchait et al., 2016).

We believe that error aversion culture may weaken the positive influence of ICT use and efficacy among new generation teachers. There are two reasons: first, when the error aversion culture is high, new generation teachers will reduce the exploration of ICT in teaching due to the fear of making mistakes. In addition, some new generation teachers who experiment with ICT find it difficult to learn from mistakes. Because in error aversion culture, they are reluctant to share their failing experiences and communicate the reasons for mistakes. New generation teachers who have unsuccessful experiences may dampen their confidence in improving student learning. Second, when error aversion culture is high, the new generation teachers need to hide mistakes or blame others in order to restore their personal image. New generation teachers seldom communicate with each other and are reluctant to share problems in ICT use so as to avoid being exposed or criticized by others. Interpersonal relationship between colleagues becomes relatively tensed. In strained interpersonal relationships, new generation teachers are distracted (Oldham, 1976) and are unable to fully participate in the classroom teaching plan and organization, which may decrease of their efficacy. On the contrary, when error aversion culture is low, new generation teachers can explore and learn independently without worrying about the risk of personal image damage caused by mistakes. New generation teachers can ICT to explore different teaching methods, which increases their possibility of providing personalized learning content. Correspondingly this may increase their sense of efficacy. When error aversion culture is low, new generation teachers may take the initiative to share their experiences of mistakes with colleagues and discuss the causes of mistakes and improvement methods so as to improve the teaching ability and effect, and enhance new generation teachers’ sense of efficacy. Therefore, we propose our third hypothesis as follows:

H3: Error aversion culture negatively moderates the relationship between ICT use and new generation teachers’ efficacy. Higher the organizational error aversion culture, weaker the positive impact of ICT use on efficacy in new generation teachers.

**FIGURE 1:**
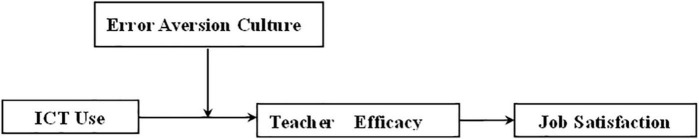
The proposed model of ICT use and job satisfaction.

The theoretical model, along with all research hypotheses labeled, is presented in Figure 1.

## Materials and Methods

### Sample

We collected data from new generation teachers in China. The literature considers teachers to belong to the new generation if they are born between 1981 and 1999 or after 1978 (Kehrli and Sopp, 2006; Borges et al., 2010). We used as a proxy the teachers aged between 22 and 43 as belonging to the new generation. To maintain the validity of the empirical data set, we included a qualification question in our online questionnaire to ensure only new generation teachers were included in the sample. To ensure the quality of the survey, we promised the participants that the data collected would only be used for academic research purposes and assured them that the questionnaire was without identity information.

We used a survey instrument to collect empirical data from primary and secondary schools of China, where teacher must participate the information technology application ability enhance project. Data were collected between May and June 2021. An online questionnaire was designed to collect data through the survey website www.credamo.com. The online surveys were based on a wide number of primary and secondary school teachers so that a sufficient number of samples can be obtained to ensure the accuracy and universality of the research. As authors were more familiar with educational information of Guangxi province, we limited our survey to this geographical area. Incomplete questionnaires were disqualified from the sample. In the end, 250 teachers received the survey invitation, and 234 completed the survey. The response rate was 83.6%, lending high confidence to the validity of finding in this research.

Description of sample is shown in Table 1. Among the respondents, 41.9% were males, 62% respondents were aged 22–30 years, while 34.6% teachers were aged 30–40 years, and 3.4% were aged 40–43 years. 73.1% of the respondents had a bachelor’s degrees, and most respondents had a more than 3 years teaching tenure.

**TABLE 1:** Sample characteristics and distribution (*N* = 234).

Demographics	Categories	Number	%
Gender	Male	98	41.9
	Female	136	58.1
Age	22–25 years	79	33.8
	25–30 years	66	28.2
	30–40 years	81	34.6
	40–43 years	8	3.4
Education	Junior college or below	19	8.1
	University	171	73.1
	Graduate school	44	18.8
Teaching tenture	Blow 3 years	89	38
	3–6 years	62	26.5
	6–9 years	61	26.1
	9–15 years	18	7.7
	15 years or above	4	1.7

### Measures

The scale used in this study are based on the literature. For most items we used existing scales with minimal adaptation. To ensure accuracy and validity of the questionnaire, we conducted a pre-survey with 10 new generation teachers and three experts in the field. New generation teacher gave some suggestions regarding the content of the questionnaire, and assured that the respondents would properly understand the questions. The experts helped to modify the questions to fit the purpose of the survey. Thus, the final form of the questionnaire was developed.

#### Information and Communication Technology Use

New generation teacher differences in ICT use were assessed by administering the scale for measuring computer use developed by Van Braak (2001). The scale has been adopted by former researcher (Sang et al., 2011). The subjects responded to the question “I will use information technology to complete classroom teaching, and also let students use computers to report homework.” New generation teachers responded to 8 items on the seven-point scale with (1) indicating “strongly disagree” and (7) indicating “strongly agree.” The scale has high levels of internal reliability, α = 0.779.

#### Job Satisfaction

We measured job satisfaction using the Tsui et al. (1992) six-item scale for employee job satisfaction. The agreement degree of new-generation teachers for these items on a seven-point scale ranges from “strongly disagree” (1) to “strongly agree” (7) (α = 0.827).

#### Teacher Efficacy

The twelve item scale of Tschannen-Moran and Hoy (2001) were adopted for efficacy measurement. New generation teachers’ responses were rated with this seven-point scale, in which (1) indicated “strongly disagree” and (7) indicated “strongly agree” (α = 0.876).

#### Error Aversion Culture

New generation teachers completed a shortened version of the Survey of Error Aversion Culture (EVC; van Dyck et al., 2005). This scale has been adapted in the Chinese context (Du et al., 2015); we used the adapted scale. New generation teachers expressed their agreement degree with the items through this seven-point scale with (1) indicating “strongly disagree” and (7) indicating “strongly agree” (α = 0.906).

#### Control Variables

We controlled several variables which may influence new generation teachers’ job satisfaction. Gender (1, “male”; 0, “female”), teaching tenure (5 grade, ranging from below 3 to more than 15 years), education level (3 grade, ranging from junior college to master) and new generation employee work value (Hou et al., 2014).

### Scale Validation

We used the Harman single factor test common method bias. Under the condition of no rotation, the variation explained by the first factor was 27.528%, below the critical value of 40%, meaning the absence of severe common method bias in the current research. In order to investigate the discriminating effects of ICT use, job satisfaction, teacher efficacy and error aversion culture in this manuscript, we performed confirmatory factor analysis. As shown in Table 2, the four-factor model was obviously superior to other alternative models, χ^2^/DF = 1.622, CIF = 0.904, TFI = 0.897, RMSEA = 0.052, indicating that these four constructs have good discriminative validity.

**TABLE 2:** Comparison of the goodness-of-fit indices of alternative models.

model	χ^2^	df	χ^2^/df	CFI	TLI	RMSEA	SRMR
Four factor model: ICT, JS, EF, and ER	793.195	489	1.622	0.904	0.897	0.052	0.059
Three-factor model: ICT, JS + EF, and ER	872.662	492	1.773	0.880	0.872	0.058	0.062
Two-factor model: ICT + ER and JS + EF	1,330.432	494	2.693	0.737	0.719	0.085	0.145
Single factor model: ICT + ER + JS + EF	1,949.873	495	3.939	0.543	0.512	0.112	0.122

*ICT, ICT use; JS, job satisfaction; EF, teachers efficacy; ER, error aversion culture.*

## Results

### Means, SD, and Correlations Among Study Variables

The mean, standard deviation and correlation coefficient of variables is shown in Table 3. ICT use (*r* = 0.538, *P* < 0.001), teachers’ efficacy (*r* = 0.673, *P* < 0.001) are positively correlated with job satisfaction, error aversion culture is in a negative correlation with job satisfaction (*r* = −0.210, *P* < 0.01) and ICT use (*r* = −0.190, *P* < 0.01). The above results are consistent with the assumptions.

**TABLE 3:** Mean, standard deviation, and correlation coefficient of all variables.

Variable	Mean	S.D.	1	2	3	4	5	6	7	8
(1) ICT use	5.609	0.689	1							
(2) Job satisfaction	5.665	0.691	0.538***	1						
(3) Teachers efficacy	5.814	0.564	0.579***	0.673***	1					
(4) Errors aversion culture	3.094	1.261	−0.190**	−0.210**	−0.124	1				
(5) Gender	0.419	0.494	0.073	0.123	0.102	0.075	1			
(6) Teaching tenure	2.085	1.049	0.273***	0.319***	0.495***	0.129*	0.096	1		
(7) Education level	2.107	0.509	0.109	0.151*	0.228***	0.251***	0.146*	0.176**	1	
(8) Work values	5.986	0.467	0.507***	0.494***	0.628***	−0.123	0.03	0.183**	0.041	1

*P < 0.05, **P < 0.01, ***P < 0.001, n = 234.

### Hypotheses Test

Hierarchical regression analyses were used to verify all hypotheses, and Baron and Kenny’s three-step method was adopted for testing the mediating effect of teachers’ efficacy. Table 4 presents the results. First, the effect of ICT use on job satisfaction was tested (H1). By controlling for the effect of gender, teaching tenure, education level and work values, ICT use was included in the regression equation (see Table 4 model 2), and the variance interpretation is significantly increased (ΔR*^2^* = 0.08). ICT use was positively related to job satisfaction (β = 0.338, *t* = 5.481, *P* < 0.001); H1 was validated. The mediating effect of teachers’ efficacy was also investigated (H2). Second, it examined the relationship between ICT use and teachers’ efficacy. It can be seen from Model 5 in Table 4 that ICT use has a positive impact on teachers’ efficacy (β = 0.211, *t* = 5.219, *P* < 0.001). Third, teacher efficacy was incorporated into the regression equation (model 3). The results showed that teachers’ efficacy was in a significantly positive correlation with job satisfaction (β = 0.619, *t* = 6.676, *P* < 0.001). The regression coefficient of ICT use and job satisfaction decreased from 0.338 to 0.208 (*t* = 3.473, *P* < 0.01), indicating that teacher efficacy partially mediated the positive correlation between ICT use and job satisfaction; H2 was validated. Additionally, variance inflation factor (VIF) was adopted for further testing the multicollinearity of variables. VIF values for all variables in the regression equation were less than 5, indicating that there was no multicollinearity between the variables.

**TABLE 4:** Results of regression analysis.

Variable	Job satisfaction			Self-efficacy		
		
	Model 1	Model 2	Model 3	Model 4	Model 5	Model 6
Control variables						
Gender	0.107	0.091	0.076	0.035	0.024	0.034
Teaching tenure	0.142***	0.103**	−0.004	0.197***	0.173***	0.189***
Education level	0.114	0.089	0.005	0.151**	0.136**	0.177***
Work value	0.664***	0.429***	0.105	0.670***	0.524***	0.521***
**Independent variables**						
ICT use		0.338***	0.208**		0.211***	0.191***
**Mediating variable**						
Teacher efficacy			0.619***			
**Moderating variable**						
Errors aversion culture						−0.045*
ICT use * error aversion culture						−0.062*
R^2^	0.312	0.392	0.492	0.564	0.61	0.627
Adjusted R^2^	0.300	0.379	0.479	0.556	0.602	0.616
*F* value	26.004**	29.449***	36.660***	73.956***	71.391***	54.363***

Non-standard regression coefficient, ***P < 0.001, **P < 0.01, *P < 0.05.

### Moderating Effect Test

**FIGURE 2:**
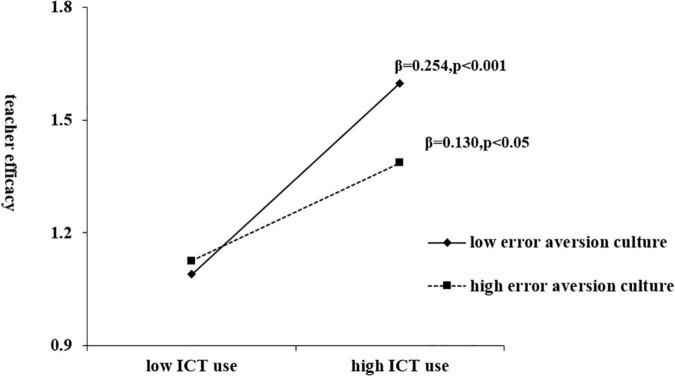
Moderating effect of error aversion culture.

In Model 6 of Table 4, teachers’ efficacy was taken as the dependent variable, and control variables, error aversion culture, ICT use and their interaction terms were added. The interaction coefficient was significant (β = −0.062, *P* < 0.05), meaning that the improvement of error aversion culture can decrease the positive effect of ICT use on teachers’ efficacy. Figure 2 constructs the relationship between ICT use and teacher efficacy under different levels of error aversion culture. According to the results of simple slope analysis, ICT use generated more positive effect on teachers’ efficacy in the case of low error aversion culture compare to high error aversion culture.

## Discussion

### Research Conclusion

The objective of this study was to understand the effect, mechanism and boundary condition of ICT use on new generation teacher work outcomes. Drawn upon the person-job fit theory, we proposed an ICT use framework to examine the interrelationship between new generation teachers, technology, school, and explore the influence of ICT use on efficacy and job satisfaction. Further, we investigated the moderating effect of error aversion culture on ICT use and efficacy. Through a cross-section survey study with 234 new generation teachers in China we found that ICT use had a positive effect on job satisfaction. Teacher efficacy is the mediator between ICT use and job satisfaction, and error aversion culture decreased the positive relationship between ICT use on teacher efficacy.

### Theoretical Contribution

This study found that job satisfaction is one of the desired outcomes of ICT utilization and new generation teachers’ fit. By exploring the mechanism and boundary conditions of how ICT use influence work outcome, this study makes several contributions to the literature on ICT effect.

Our research complements extant research focusing on the association between ICT use and job satisfaction by introducing person-job fit theory. Our findings facilitate an understanding of the complex mechanism underlying the relationship between ICT use and job satisfaction. The mediating effects of teacher efficacy were empirically examined. Previous studies have investigated the mediating effects of burnout. However, based on the person-job fit theory, individual characteristics and its fit with job have been greatly ignored. Unlike prior studies considering the perspective of technostress and job characteristics (Tarafdar et al., 2010, 2014; Venkatesh et al., 2010; Srivastava et al., 2015; Ninaus et al., 2021), the person-job fit theory incorporated the interaction between ICT and new generation teachers. Findings of this study indicating that ICT use increase new generation teachers’ job satisfaction, Venkatesh et al. (2010) claimed that employees have difficulties in adapting to ICT use and therefore, their job satisfaction decreases. Teacher efficacy plays a partial mediating role in ICT use and job satisfaction, this result is consistent with previous researches that person-job fit increases individual self-efficacy, thereby enhancing job satisfaction.

Further, this study extends existing ICT use literature by focusing on organizational culture context. Prior studies have argued that culture influences person-environment fit (Chuang et al., 2015). Few studies have explored how organizational culture influences the fit of individuals and ICT on individual outcomes; and the underline mechanism remains unclear. Our study proposes that ICT use by new generation teachers is a trial and error process. Different from extant studies which mainly focused on the school infrastructure and leadership (Andersen, 2016; Gil-Flores et al., 2017); we empirically examined the roles of error averse culture in changing teachers ICT use efficacy. In doing so, this study complements the understanding of mechanisms about organizational culture on ICT use.

### Limitations and Future Research Prospects

This study has some limitations. First, although we conducted analyses to check for common method bias, longitudinal research design or multiple responder data collection could eliminate possible bias. Second, we collected data from the new generation teachers of primary and second schools in Guangxi province of China. The sampling is based on the principle of convenience, which could reduce the generalizability of our findings. In future studies, data should be collected from diverse regions to investigate the effect of ICT use by new generation teachers. Future studies could focus on other generation teachers’ ICT use as well. Third, although psychological emotions play a potential role in ICT use, we did not examine the relationship between ICT use and psychological emotions. Future studies could investigate the effect of ICT use on teachers’ psychological emotions to enrich ICT use research.

## Data Availability Statement

The original contributions presented in this study are included in the article/supplementary material, further inquiries can be directed to the corresponding author.

## Author Contributions

PX designed the study, performed the research, analyzed data, and wrote the initial draft of the manuscript. YJ contributed to refining the ideas, carrying out additional analyses, and finalizing the manuscript. Both authors contributed to the article and approved the submitted version.

## Conflict of Interest

The authors declare that the research was conducted in the absence of any commercial or financial relationships that could be construed as a potential conflict of interest.

## Publisher’s Note

All claims expressed in this article are solely those of the authors and do not necessarily represent those of their affiliated organizations, or those of the publisher, the editors and the reviewers. Any product that may be evaluated in this article, or claim that may be made by its manufacturer, is not guaranteed or endorsed by the publisher.
